# m-CT值在预测临床Ia期肺癌和癌前病变恶性程度中的应用研究

**DOI:** 10.3779/j.issn.1009-3419.2018.03.13

**Published:** 2018-03-20

**Authors:** 汉然 吴, 常青 柳, 美青 徐, 燃 熊, 广文 徐, 彩伟 李, 明然 解

**Affiliations:** 230001 合肥，中国科学技术大学附属第一医院胸外科 Department of Toracic Surgery, the First Afliated Hospital of University of Science and Technology of China, Hefei 230001, China

**Keywords:** 肺肿瘤, 体层摄影术, X线计算机, ROC曲线, Lung neoplams, Tomography, X-Ray computed, ROC curve

## Abstract

**背景与目的:**

近年来，肺部磨玻璃结节（ground-glass opacity, GGO）的检出率逐年升高，预测GGO的恶性程度对临床治疗至关重要。本研究旨在评价m-CT值（mean computed tomography value, m-CT）在预测早期肺癌和癌前病变恶性程度中的价值，并将m-CT值与MaxCT值（Max computed tomography value, MaxCT）、GGO直径、GGO实性成分直径及C/T比值（consolid/tumor ratio, C/T）对比，得出最佳影像学评价指标。

**方法:**

回顾性分析中国科学技术大学附属第一医院胸外科2012年1月-2013年12月接受肺切除手术并有病理证实的肺GGO患者129例，其中非典型腺瘤样增生（atypical adenomatous pyperplasia, AAH）43例、原位腺癌（adenocarcinoma *in situ*, AIS）26例、微浸润腺癌（minimally invasive adenocarcinoma, MIA）17例、腺癌43例。将AAH和AIS归为非浸润（noninvasive cancer, NC）组，69例，MIA和腺癌归为浸润（invasive cancer, IC）组，共60例。通过绘制受试者工作曲线得出实性成分大小、肿瘤大小、C/T比值、m-CT值和Max CT值的cutoff值和曲线下面积，再通过单因素和多因素分析临床资料和CT参与与GGO恶性程度的相关性。

**结果:**

本研究共入组了129例患者，其中男性59例，女性70例，平均年龄（62.0±8.6）岁。两组患者在性别、年龄、分化程度方面没有差异无统计学意义（*P* > 0.05）。通过绘制患者的实性成分大小、肿瘤大小、C/T比值、m-CT值和Max CT值的ROC曲线发现，上述指标的cutoff值分别为：9.4 mm、15.3 mm、47.5%、-469.0 HU和-35.0 HU，对应的AUC分别为：0.89、0.79、0.82、0.90、0.85，m-CT值的曲线下面积最大，对预测GGO恶性程度效果最佳。单因素和多因素分析发现，肿瘤大小、C/T比值、m-CT值和Max CT值均与GGO恶性程度有较强的相关性。

**结论:**

GGO大小、GGO中实性成分大小、Max CT值、m-CT值、C/T比值与GGO的恶性程度均有一定的相关性。m-CT值预测纯GGO的恶性程度的准确性相对最高，而对于混合密度的GGO病变，需要结合m-CT值、Max CT值及GGO大小来综合评估。

## Abstract (en)

**Background and objective:**

Recently, the detectable rate of ground-glass opacity (GGO) was signifcantly increased, a appropriate diagnosis before clinic treatment tends to be important for patients with GGO lesions. Te aim of this study is to validate the ability of the mean computed tomography (m-CT) value to predict tumor invasiveness, and compared with other measurements such as Max CT value, GGO size, solid size of GGO and C/T ratio (consolid/tumor ratio, C/T) to fnd out the best measurement to predict tumor invasiveness.

**Methods:**

A retrospective study was conducted of 129 patients who recieved lobectomy and were pathological confrmed as atypical adenomatous pyperplasia (AAH) or clinical stage Ia lung cance in our center between January 2012 and December 2013. Of those 129 patients, the number of patients of AAH, AIS, AIS and invasive adenocarcinoma were 43, 26, 17 and 43, respectively. We defned AAH and AIS as noninvasive cancer (NC), MIA and invasive adenocarcinoma were categorized as invasive cancer(IC). We used receiver operating characteristic (ROC) curve analysis to compare the ability to predict tumor invasiveness between m-CT value, consolidation/tumor ratio, tumor size and solid size of tumor. Multiple logistic regression analyses were performed to determine the independent variables for prediction of pathologic more invasive lung cancer.

**Results:**

129 patients were enrolled in our study (59 male and 70 female), the patients were a median age of (62.0±8.6) years (range, 44 to 82 years). Te two groups were similar in terms of age, sex, differentiation (*P* > 0.05). ROC curve analysis was performed to determine the appropriate cutoff value and area under the cure (AUC). Te cutoff value of solid tumor size, tumor size, C/T ratio, m-CT value and Max CT value were 9.4 mm, 15.3 mm, 47.5%, -469.0 HU and -35.0 HU, respectively. Te AUC of those variate were 0.89, 0.79, 0.82, 0.90, 0.85, respectively. When compared the clinical and radiologic data between two groups, we found the IC group was strongly associated with a high m-CT value, high Max CT value, high C/T ratio and large tumor size. Gender, solid tumor size, tumor size, C/T ratio, m-CT value and MaxCT value were selected factor for multivariate analysis, when using the preoperatively determined variables to predict the tumor invasiveness, revealed that tumor size, C/T ratio, m-CT value and Max CT value were independent predictive factors of IC.

**Conclusion:**

Te musurements of Max CT value, GGO size, solid size of GGO and C/T ratio were signifcantly correlated with tumor invasiveness, and the evaluation of m-CT value is most useful musurement in predicting more invasive lung cancer.

肺癌在全世界范围内发病率和死亡率居所有恶性肿瘤之首^[1, 2]^。近年来随着影像学技术的发展和低剂量螺旋计算机断层扫描(computed tomography, CT)体检的普及，肺部磨玻璃结节(ground-glass opacity, GGO)的检出率逐年升高。GGO好发于双肺外周或胸膜下，CT肺窗表现为肺部局灶密度增高的云雾状区域，内部血管和支气管未受侵犯。临床上绝大多数GGO为早期肺癌或癌前病变，包括：非典型腺瘤样增生(atypical adenomatous hyperplasia, AAH)、原位腺癌(adenocarcinoma *in situ*, AIS)及微浸润腺癌(minimally invasive carcinoma, MIA)，这三种病变均较浸润性腺癌预后更好^[3, 4]^。近期研究^[5, 6]^表明，仅对这部分患者进行亚肺叶切除，就能获得良好的预后。因此，术前预测GGO的恶性程度，避免临床上过度的外科手术切除非常重要。

目前临床上尚无评判GGO恶性程度的统一标准，临床上常根据GGO的影像学特征如形态、边缘、界面、内部结构、血管集束征、胸膜凹陷征等来预测其恶性程度，误诊率相对较高。本研究回顾性分析了129例术前诊断GGO、接受肺切除手术且术后病理证实为腺癌或癌前病变的患者的临床相关资料和CT参数(GGO的直径、C/T比值、Max CT值和m-CT值)，评价上述CT参数与GGO恶性程度的相关性。

## 1 资料与方法

### 1.1 一般资料

收集中国科学技术大学附属第一医院胸外科2012年1月-2013年12月接受肺切除手术并有病理证实的肺GGO患者129例，其中男性59例，女性70例，平均年龄(62.0±8.6)岁。患者的病理类型根据最新的国际肺癌协会、美国胸科学会、欧洲呼吸学会联合发布的肺腺癌的国际多学科分类标准分为：A AH、AIS、MIA。将A AH和AIS归为非浸润(noninvasive cancer, NC)组，MIA和腺癌归为浸润(invasive cancer, IC)组。患者的术前检查包括：血常规、生化、凝血象、免疫组合，心电图、超声心动图，肺功能，气管镜检查，同时行胸部平扫+增强CT+肺部GGO三维重建明确病变性质，行腹部+双侧肾上腺彩超、头颅MRI平扫+增强、骨扫描检查以排除远处转移。

### 1.2 方法

#### 1.2.1 患者纳入与排除标准

纳入标准：(1)行高分辨率CT检查提示肺部外周型孤立性结节，直径小于2 cm，病理证实为腺癌或癌前病变；(2)气管镜检查提示段及段以上支气管无病变；(3)术前分期为cT_1_N_0_M_0_；(4)手术方式为肺段切除、肺叶切除及肺楔形切除。排除标准：(1)术前检查提示有淋巴结肿大(CT上直径≥1 cm)或正电子发射型断层显像(positron emission tomography, PET-CT)怀疑有淋巴结转移；(2)术前接受肺穿刺活检，已明确病理类型的患者；(3)临床资料不完整。

#### 1.2.2 方法

临床资料分析：所有患者的临床资料均来自我院病案室，统计患者的年龄、性别、术前cTNM分期，术后TNM分期，病理类型，手术方式。CT扫描及图像分析：收集所有患者的CT扫描结果。设备为Simens Somatom Sensation 64层螺旋CT机，行CT检查时嘱患者在吸气末屏气用5 mm层面的薄层CT机从胸廓入口扫面到肺底部。扫描参数为：120 kV，80 mA-160 mA，螺距1.375，进床速度为27.5 mm/r，扫描FOV 30 cm-35 cm。需要行三围重建的CT图像采用层厚1.25 mm-1.5 mm，层距1 mm-1.5 mm进行图像重建。观察窗位为：肺窗窗宽1, 500 HU-2, 000 HU (Hounsfied units, HU)，窗位-700 HU-600 HU，纵隔窗窗宽350 HU-400 HU，窗位35 HU-50 HU。

CT数据采集由两位工作高年资影像科医师在未知患者病理结果的情况下采集，将肺部外周型孤立性结节阴影完全为云雾状、气管和血管无外侵且在HRCT中无实性成分的归为GGO。在行三维重建时应用A DW3.1 (Advantage Workstation 4.3; GE Healthcare)，利用重建软件(Lung VCAR; GE Healthcare)进行三维重建。采集GGO的影像学数据应用View Pro-X软件进行分析，包括：病灶位置、GGO最大径、GGO中实性成分最大径、最大CT值、平均CT(m-CT)值、C/T比。两位高年资医师得出的上述数据出现分歧时通过计算平均值得出结果。

### 1.3 统计学方法

采用SPSS 18.0统计学软件对数据进行分析。正态分布资料用(Mean±SD)表示，计量资料均数比较采用*t*检验，计数资料比较采用卡方检验。应用单因素和多因素分析来研究患者的临床资料，计算95%的可信区间，单因素分析中*P* < 0.05的指标结合文献报道后纳入多因素分析，并绘制GGO最大径、实性成分最大径、m-CT、C/T比值等的受试者工作特征曲线(receiver operating characteristics, ROC)，同时计算曲线下面积(area under the curve, AUC)和cut-off值，AUC在0.5-0.7之间为诊断价值较低，0.7-0.9为诊断价值中等，> 0.9诊断价值价高。以*P* < 0.05为差异有统计学意义。

## 2 结果

### 2.1 患者的临床资料

本研究共入组了129例患者，其中男59例，女70例，平均年龄(62.0±8.6)岁。患者的TNM分期、手术方式、分化程度和IASLC分类情况见表 1。

**1:** 患者临床病理特征（*n*=129） Patient clinical and pathologic characteristics (*n*=129)

Factors	*n* (%)
Gender	
Male	59(45.74)
Female	70(54.26)
Surgical approach	
Wedge resection	41(31.78)
Segmentectomy or lobectomy	88(68.21)
pT category	
T1	102(79.1)
T2	27(20.9)
pN category	
N0	109(84.5)
N1	20(15.5)
pTNM stage	
Ia	106(82.17)
Ib	3(2.33)
IIa	20(15.5)
pG category	
G1	54(41.86)
G2	66(51.16)
G3	9(6.98)
IASLC classification	
AAH	43(33.33)
AIS	26 (20.16)
MIA	17 (13.18)
Invasive adenocarcinoma	43 (33.33)
IASLC: International Association for the Study of Lung Cancer; AIS: adenocarcinoma *in situ*; MIA: Minimally invasive adenocarcinoma.	

### 2.2 两组患者的临床资料和CT参数的比较

两组患者在性别、年龄、分化程度方面没有差异无统计学意义(*P* > 0.05)(表 2)。通过绘制患者的实性成分大小、肿瘤大小、C/T比值、m-CT值和Max CT值的ROC曲线发现(图 1)，上述指标的cutoff值分别为：9.4 mm、15.3 mm、47.5%、-469.0 HU和-35.0 HU，对应的AUC分别为：0.89、0.79、0.82、0.90、0.85，对应的95%CI分别为：0.826-0.959、0.706-0.868、0.738-0.906、0.851-0.954、0.788-0.915。上述指标中m-CT值的AUC最大，对评估GGO的恶性程度效果最佳。根据文献报道的结果结合本文纳入的指标将患者的性别、实性成分大小、肿瘤大小、C/T比值、m-CT值和Max CT值纳入单因素分析发现，实性成分大小、肿瘤大小、C/T比值、m-CT值和Max CT值与GGO的恶性程度存在相关性(*P* < 0.001，表 3)，通过多因素分析发现，肿瘤大小、C/T比值、m-CT值和Max CT值均GGO恶性程度的独立危险因素(表 4)。

**2:** 两组患者临床资料和CT参数间的比较 Comparison of clinical and radiologic data between lesions in NC and IC

Factors	Noninvasive cancer group (*n*=69)	Invasive cancer group (*n*=60)	*X*^2^/*t*	*P*
Gender			1.087	0.297
Male	35(50.7%)	24(40.0%)		
Female	34(49.3%)	36(60.0%)		
Age(yr)	58.48±8.23	60.97±8.98	0.330	0.567
pG category			13.278	0.001
G1	38(55.1%)	16(26.7%)		
G2	25(36.2%)	41(68.3%)		
G3	6(8.7%)	3(5.0%)		
Solid tumor size (mm)			53.04	< 0.001
≤9.4	59(85.5%)	13(21.7%)		
> 9.4	10(14.5%)	47(78.3%)		
Tumor size (mm)			37.91	< 0.001
≤15.3	62(89.9%)	23(38.3%)		
> 15.3	7(10.1%)	37(61.7%)		
C/T ratio (%)			39.18	< 0.001
≤47.5	60(87.0%)	20(33.3%)		
> 47.5	9(13.0%)	40(66.7%)		
m-CT value (HU)			58.45	< 0.001
≤-469.0	61(88.4%)	13(21.7)		
> -469.0	8(11.6%)	47(78.3)		
Max CT value (HU)			50.25	< 0.001
≤-35.0	51(73.9%)	7(11.7%)		
> -35.0	18(26.1%)	53(88.3%)		

**1:**
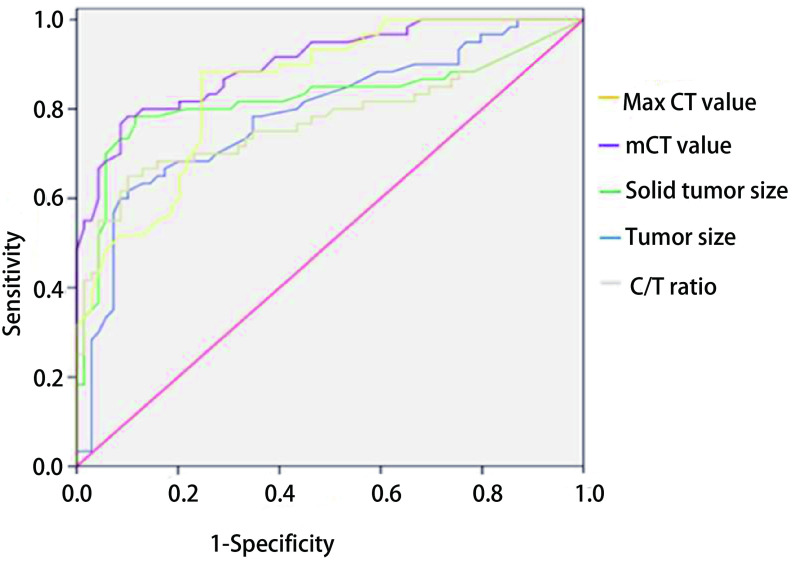
CT参数的ROC曲线 Receiver operating characteristics (ROC) curves predicting GGO invasiveness. GGO: ground-glass opacity.

**3:** 预测sGGO恶性程度的临床资料和CT参数的单因素回归分析 Univariate *Logistic* regression analysis predicting the GGO invasiveness

Factors	Odds ratio	95%CI	*P*
Gender			0.223
Male *vs* Female	1.54	0.77-3.11	
pT category			< 0.001
T1a *vs* T1b	> 999.999	< 0.001- > 999.999	0.941
T1a *vs* T2a	> 999.999	< 0.001- > 999.999	0.926
T1a *vs* T2b	> 999.999	< 0.001- > 999.999	0.948
pN category			0.556
N0 *vs* N1	1.324	0.519-3.379	
pTNM stage			< 0.001
Ia *vs* Ib	> 999.999	< 0.001- > 999.999	0.983
Ia *vs* IIa	9.35	2.58-33.92	< 0.001
pG category			0.086
G1 *vs* G2	3.89	1.81-8.39	< 0.001
G1 *vs* G3	1.19	0.26-5.34	0.823
IASLC classification			< 0.001
AAH *vs* AIS	1	< 0.001- > 999.999	1
AAH *vs* MIA	> 999.999	< 0.001- > 999.999	0.52
AAH *vs* Invasive adenocarcinoma	> 999.999	< 0.001- > 999.999	0.393
Solid tumor size (mm)			< 0.001
≤9.4 *vs* > 9.4	0.047	0.019-0.116	
Tumor size (mm)			< 0.001
≤15.3 *vs* > 15.3	0.07	0.027-0.18	
C/T ratio (%)			< 0.001
≤47.46 *vs* > 47.46	0.075	0.031-0.181	
m-CT value (HU)			< 0.001
≤-469 *vs* > -469	0.036	0.014-0.095	
Max CT value (HU)			< 0.001
≤-35 *vs* > -35	0.047	0.018-0.121	

**4:** 预测sGGO恶性程度的临床资料和CT参数的多因素回归分析 Multiple *Logistic* regression analysis predicting the GGO invasiveness

Factors	Odds ratio	95%CI	*P*
Gender			0.422
Male *vs* Female	2.380	0.286-19.784	
Solid tumor size (mm)			0.666
≤9.4 *vs* > 9.4	0.594	0.056-6.318	
C/T ratio (%)			< 0.001
≤47.46 *vs* > 47.46	0.030	0.003-0.348	
Max CT value (HU)			< 0.001
≤-35 *vs* > -35	0.020	0.001-0.314	
M-CT value (HU)			< 0.001
≤-469 *vs* > -469	0.049	0.006, 0.380	
Tumor size (mm)			0.023
≤15.3 *vs* > 15.3	0.046	0.003-0.653	

## 3 讨论

近年来随着影像学技术的快速发展，GGO的检出率也越来越高^[7-10]^。目前对于GGO的处理主要包括定期随访和手术切除，临床上常根据GGO的倍增时间和影像学特征来预测其恶性程度。但仅根据影像学特征来判断GGO的恶性程度与阅片医师的经验相关较大，误诊率较高，导致部分只需要接受亚肺叶切除手术的患者接受了更大范围手术治疗，部分需要手术切除的患者在随访过程当中错过了最佳手术时机。近年来，各种CT参数在GGO诊断中的应用逐渐被重视，特别是通过对C/T比值、m-CT值、Max CT值、GGO及GGO中实性成分大小的研究发现，上述指标与GGO的恶性程度关系密切^[11, 12]^。本研究发现，GGO的大小及其实性成分大小、m-CT值及Max CT值与GGO的恶性程度均有显著相关性，其中m-CT值的评估价值最高。

2016年美国国立综合癌症网络(National Comprehensive Cancer Network, NCCN)发布的肺癌筛查指南中指出，对于实性成分 > 5 mm的混合GGO或直径 > 10 mm的纯GGO，根据其风险程度怀疑恶性病变的建议活检或手术切除^[13]^。本研究发现，GGO最大径、实性成分最大径与GGO的恶性程度密切相关，当GGO的直径> 15.3 mm时或GGO中实性成分 > 9.4 mm时，倾向为浸润性病变，建议及时的外科干预治疗。有研究^[7]^通过对188例GGO患者的CT资料进行分析发现，不同病理类型的GGO的直径存在差异，并且认为当GGO直径> 15.5 mm GGO的恶性程度较高，建议手术切除。Lee等^[11]^通过对纯GGO的病理类型和CT资料研究发现，当GGO的直径>15 mm时GGO倾向于浸润性腺癌。Tamura等^[12]^报道当GGO > 15.6 mm、GGO中实性成分 > 9 mm时，GGO的恶性程度较高。目前文献报道的GGO的直径的临界值差别不大，均在15 mm左右。本研究中GGO直径的阈值为15.3 mm，与文献^[7, 11, 12]^报道的类似。

本研究发现，C/T比值与混合型GGO的恶性程度具有显著相关性。GGO中实性成分的主要病理改变为：(1)肺间质的纤维炎性增生和炎性细胞浸润；(2)表现为层叠排列的癌细胞，肺泡结构不同程度受损且在致密的纤维结构中存在肿瘤细胞浸润。周边毛玻璃样变的主要成分为出血和炎症反应。所以理论上GGO的肿瘤成分越多，GGO的C/T比值越大，恶性程度也相对较高。美国胸科医师学会(American College of Chest Physicians, ACCP)在2013年修订的肺癌诊治指南^[14]^中指出，当混合密度GGO在随访过程中实性成分比例增大(> 50%)，GGO的恶性程度较高。Tamura等^[12]^通过对494例GGO患者的C/T比值进行研究发现，当GGO中C/T比值> 50.4%时，GGO倾向为浸润性病变。本研究中通过绘制ROC曲线计算临界值发现，C/T比值大于47.5%时，GGO的恶性程度较高，此时外科切除是最佳选择。

目前关于m-CT值与GGO恶性程度的研究报道较少^[11, 12]^，本研究中通过绘制GGO直径、GGO实性成分直径、C/T比值、m-CT值的ROC曲线发现，m-CT值的AUC最大，以m-CT值来评估GGO恶性程度价值最高。当m-CT大于-469 HU时，GGO倾向为浸润性病变。Tamura等^[12]^研究发现，m-CT值对评估GGO的恶性程度有重要意义，同时通过绘制ROC曲线发现，区分GGO浸润性的m-CT值临界值为-445 HU。该研究认为对于纯GGO病变，m-CT的评估价值最高；而对于混合密度的GGO，需要同时结合m-CT值和C/T比值来进一步诊断。Ikeda等^[15]^通过分析33例GGO患者的CT值发现，m-CT值对鉴别GGO的良恶性价值较高，同时得出AAH和AIS的鉴别临界值为-584 HU，AIS和肺腺癌的鉴别临界值为-472 HU。Peng等^[16]^通过对160例GGO患者的三维CT进行分析，通过绘制ROC曲线得出结论：m-CT值鉴别GGO中NC和MIA的临界值为-615.5 HU，鉴别MIA和腺癌的临界值为-464 HU。目前m-CT值对鉴别GGO恶性程度的意义已被文献证实^[11, 12, 15, 16]^，但对m-CT值的临界值各中心报道不一，主要在-490 HU-440 HU之间，本研究中m-CT值的临界值为-469 HU，与文献报道相似。我们认为，对于纯GGO病变，以m-CT值来评估恶性程度最佳，对于混合密度的GGO，同时结合m-CT值、Max CT值及GGO大小来综合评估准确性更高。

综上所述，综合应用GGO大小、GGO中实性成分大小、MaxCT值、m-CT值、C/T比值预测GGO的恶性程度较为可靠，其中m-CT值预测纯GGO的恶性程度相对最为准确。当纯GGO的m-CT值 > -469 HU时，GGO病灶恶性程度较高，建议及时外科手术治疗，当m-CT值 < -469HU时，可建议暂时随访观察。而对于混合密度的GGO病变，需要结合m-CT值、Max CT值及GGO大小来综合评估。由于本研究是单中心回顾性分析，存在一定病例选择性偏倚，随访时间相对较短，虽然近期效果明显，远期效果有待进一步验证。
